# High-speed crystal detection and characterization using a fast-readout detector

**DOI:** 10.1107/S0907444910028192

**Published:** 2010-08-13

**Authors:** Jun Aishima, Robin L. Owen, Danny Axford, Emma Shepherd, Graeme Winter, Karl Levik, Paul Gibbons, Alun Ashton, Gwyndaf Evans

**Affiliations:** a Diamond Light Source, Harwell Science and Innovation Campus, Didcot OX11 0DE, England

**Keywords:** macromolecular crystallography, microfocus, grid scan, sample characterization

## Abstract

A novel raster-scanning method combining continuous sample translation with the fast readout of a Pilatus P6M detector has been developed on microfocus beamline I24 at Diamond Light Source. This fast grid-scan tool allows the rapid evaluation of large sample volumes without the need to increase the beam size at the sample through changes in beamline hardware. A slow version is available for slow-readout detectors. Examples of grid-scan use in centring optically invisible samples and in detecting and characterizing numerous microcrystals on a mesh-like holder illustrate the most common applications of the grid scan now in routine use on I24.

## Abstract 2

A grid-scan tool that enables rapid characterization of large sample volumes using a microfocused X-ray beam and a fast-readout detector is reported.

## 1. Introduction   

Developments such as robotic sample changers, fast CCD and pixel-array detectors and automated data reduction have greatly increased the throughput of traditional macromolecular crystallography (MX) experiments for large crystals (dimensions ∼50 × 50 × 50 µm and greater) and similarly sized beams. More recently, microfocus X-ray instruments have enabled the measurement of high-quality diffraction data from crystal samples that were hitherto deemed to be too small or too disordered, reducing the size of crystals required for successful structure solution (Riekel *et al.*, 2005 ▸). Unfortunately, the combination of small beam sizes and small crystals, coupled with poor optical properties of samples, have conspired to make sample alignment and detection more difficult in microbeam diffraction experiments.

In this short communication, we describe a tool designed for users of the microfocus beamline I24 at Diamond Light Source that enables the straightforward and fast location of crystals and diffraction characterization of sample holders, thereby fully exploiting the advantages offered by a microfocus beamline and greatly increasing the throughput of I24. This grid-scan tool has now been in routine use for more than a year on I24 and results illustrating its power and usefulness in rapid crystal detection and evaluation are presented.

## 2. Methods   

### 2.1. Beamline I24, Diamond Light Source   

I24 is a tuneable microfocus beamline currently configured to allow users to select one of three beam sizes at the sample, 8 × 8, 20 × 20 or 50 × 30 µm (horizontal × vertical FWHM), which is achieved by focusing two pairs of KB Bimorph mirrors (Evans *et al.*, 2006 ▸). An X-­ray energy of 12.68 keV and a beam size of 8 × 8 µm were used for the experiments reported here. Diffraction images were collected with either a Rayonix MX300 CCD (Rayonix LLC, Evanston, Illinois, USA) or a Pilatus P6M (Dectris Ltd, Baden, Switzerland) detector.

### 2.2. Grid scanning   

Microfocus beamline users generally face one of two problems prior to successful data collection: (i) the location of a small microcrystal(s) within a larger sample mount and alignment of these with the X-ray beam or (ii) the location of a well diffracting crystal sub-volume in larger disordered crystals.

The problem of establishing sample location arises because crystals may be embedded within opaque material, rendering them invisible, as for crystals grown in and harvested from lipid mesophase (Caffrey, 2000 ▸), or refractive material (lens-like solvent drops), resulting in the sample appearing to be shifted from its true location. In both cases the use of X-rays for alignment of the crystal to the X-ray beam is a more reliable method than visible optics (Song *et al.*, 2007 ▸; Cherezov *et al.*, 2009 ▸).

As pioneered by Song and coworkers, our approach uses a series of diffraction images recorded at relatively low X-ray flux from different parts of the sample. In our implementation, a virtual grid is superimposed over all or part of the sample when viewed by an on-axis microscope. In the simplest case, a diffraction image is acquired at the centre of each grid box while the sample is stationary (hereafter referred to as a slow scan).

### 2.3. High-speed grid scanning   

Implementation of a faster grid scan is made possible by the P6M detector, which has a readout time of 2.3 ms, thus obviating the need to open and close the X-ray shutter between images. While this has previously been exploited to speed up conventional data-collection experiments (Hülsen *et al.*, 2006 ▸), we use the continuous detector readout together with a continuous constant-velocity sample translation to create a high-speed scanning method, where in each row of a grid a sequence of images is recorded in a single movement and exposure to the X-ray beam (a fast scan). Vertically displacing the sample between successive row measurements quickly builds up a complete grid. Vertical translations are achieved through two Attocube (Attocube Systems AG, Munich, Germany) stages mounted on the rotation stage. These are piezo-drive stepper motors and movements of submicrometre precision are possible.

A fast scan requires synchronization between the fast X-ray shutter, detector and a motion axis in order to ensure that the sample is illuminated at the positions seen on the viewing system and for the expected period of time. During a conventional diffraction experiment at I24, a TTL pulse is sent to both the fast X-ray shutter and the P6M detector to start data collection, so an accurate synchronization protocol between this pair of objects and the goniometer table *x* axis has been developed for fast grid scans. Sample translation is achieved through a servo motor controlled by a Delta Tau Turbo PMAC2 with 100 nm resolution encoder feedback. Scanning in this manner is much faster than the conventional slow scan in which images from a stationary sample are recorded following a translation.

### 2.4. Implementation   

The grid scan has been implemented in the *Generic Data Acquisition* software (*GDA*; http://www.opengda.org) using the Jython scripting language with a Java Swing graphical user interface (GUI). Control of the underlying motors and P6M detector are performed in *GDA via EPICS*. User control of the grid scan is achieved entirely through the GUI. A rectangular sample area to be scanned over is selected with a single mouse click-and-drag movement, with the horizontal and vertical pitch of the grid set to user-defined values *via* the GUI. The pitch of the grid defines the separation of adjacent scanning rows and columns and is typically set to match the beamsize, but may be increased for coarser sampling of large crystals. During the diffraction experiment this virtual grid remains overlaid on the GUI, moving with the sample and reflecting the progress of the scan. Following completion of the grid scan and user evaluation of diffraction scores or the images themselves, the sample can be centred by clicking on the box corresponding to the optimal position.

### 2.5. Image scoring and experiment feedback   

After each diffraction image has been collected, the virtual grid is updated with a local colour change indicating that a box has been exposed whilst the image is processed with *DISTL* (Zhang *et al.*, 2006 ▸) on a multi-CPU cluster. On completion of data processing, image results are stored in the ISPyB database (Beteva *et al.*, 2006 ▸) and echoed through the progress terminal to the user. As the scan progresses, the virtual grid is overlaid with coloured circles whose diameter is proportional to the normalized magnitude of the *DISTL* output criteria selected by the user. Users can select between the number of total spots, number of Bragg candidates, resolution, number of ice rings, number of overloaded spots and saturation of the strongest peaks. Results are typically displayed on the GUI within 5 s of the image being acquired.

## 3. Results and discussion   

### 3.1. Slow grid scan   

**Figure 1:**
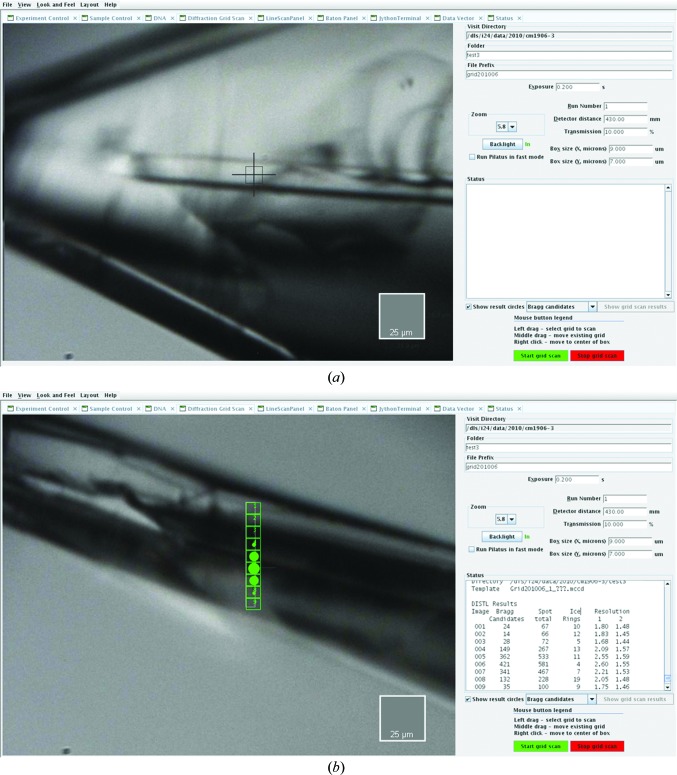
(*a*) The GUI allows the user to define the horizontal and vertical pitch of the grid (‘Box size’ fields) and data-collection parameters such as exposure time and crystal-to-detector distance. (*b*) shows the same crystal at φ + 90°; optical alignment is not possible. Diffraction data from a 1 × 7 grid have been collected and analysed with *DISTL*. *DISTL* results are automatically overlaid on the sample view and the scoring criteria can be chosen by means of a pull-down menu. Right-clicking on any box centres the sample to that position.

Fig. 1 ▸ illustrates a common application of the grid-scan tool and shows a crystal of a soluble protein complex (kindly provided by R. Steiner, King’s College London). In Fig. 1 ▸(*a*) the plane of the loop is face-on and the crystal is easily centred. In contrast, when the sample is rotated by 90° a combination of lensing effects and the opaqueness of the loop conspire to make the crystal impossible to locate visually (Fig. 1 ▸ *b*). In such cases the grid scan, coupled with automatic image scoring, rapidly establishes the crystal position. The ability to change the diffraction-quality criteria used has proved to be useful, as in our experience no single indicator is universally successful at locating crystals. This is particularly true for small crystals (dimensions of <10 µm) and visual inspection of the diffraction images is still essential in many cases. Optimizations are under way to provide improved criteria for automated evaluation.

### 3.2. Fast scan   

Synchronization tests examining the shutter-opening time and the position of the horizontal motion axis show that the average starting position of each row in a two-dimensional grid varies by less than 10% of the beam size regardless of the beam size and exposure time. Thus, the grid overlaid on the sample accurately represents the positions from which diffraction data were obtained. In future, the synchronization will be further improved by using the goniometer table *x*-axis encoder signal to trigger the shutter and detector.

**Figure 2:**
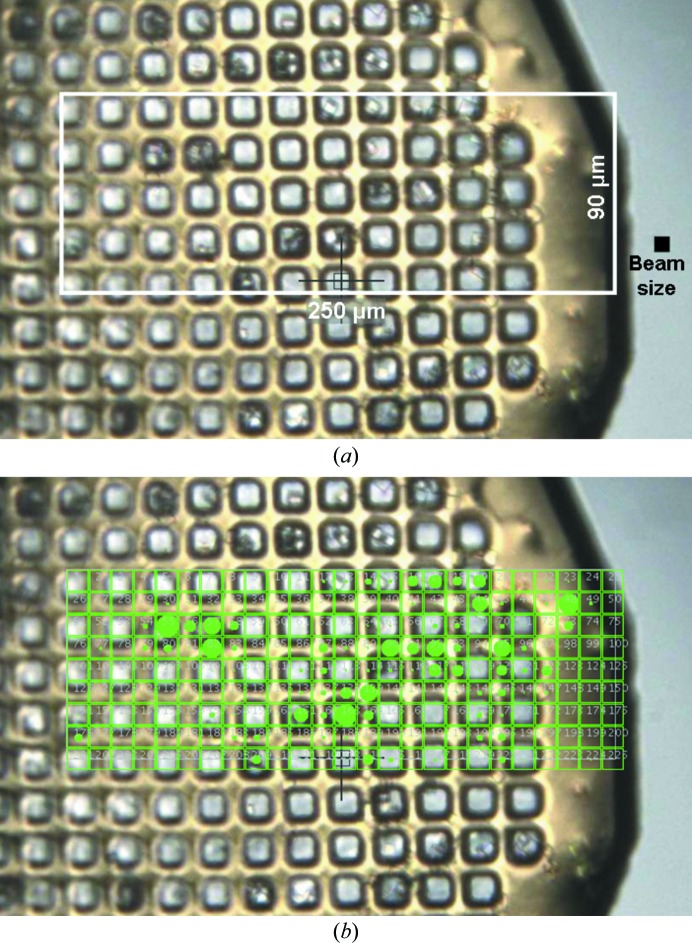
AcMNPV polyhedra crystals mounted on a MiTeGen MicroMesh. The 25 × 9 grid scan is recorded over a 250 × 90 µm region of the sample. (*a*) and (*b*) show the crystals and measurement grid without and with the *DISTL* results overlaid.

Fast grid scans enable experiments that would previously have been deemed too time-consuming or that require changes in beamline hardware. One important application is the ability to search for and evaluate crystals over entire meshes in cases where samples cannot be located visually or are small and widely distributed over a large sample holder. An example of this is shown in Fig. 2 ▸, in which a 25 × 9 grid with 10 µm pitch was measured using the fast scan across a sample of AcMNPV polyhedra crystals (Ji *et al.*, 2010 ▸) dispersed across a MiTeGen MicroMesh (MiTeGen LLC, Ithaca, New York, USA). The full I24 beam flux of ∼10^12^ photons s^−1^ was used with 1 s exposure times for the 225 images. The scan took 294 s from initiation to the presentation of all *DISTL* results as shown in Fig. 2 ▸(*b*). An equivalent grid measurement using slow scanning took 629 s. Thus, the overheads for the fast and slow scans are 69 and 404 s, respectively. At a shorter 0.2 s exposure time the fast scan took 99 s, while the slow scan took 455 s (overheads of 54 and 410 s, respectively), illustrating the greater speed gains using the fast scan with shorter exposure times.

## 4. Conclusions and future directions   

The grid-scan tool described above is in routine use on I24 and has proven to be invaluable in the detection and alignment of both microcrystals and well diffracting subvolumes of crystals. The recent implementation of a fast grid scan, exploiting the short readout time of the Pilatus detector, enables the rapid coverage of large crystal volumes or sample mounts using a microbeam. This increased speed reduces the need to first perform a coarse grid scan with larger beam size prior to a fine grid scan with a microbeam. This minimizes the need to make changes to the beamline hardware (focusing mirrors or apertures) between the crystal-detection and data-collection stages of the experiment, thereby increasing sample throughput on the beamline. Use of the fast grid scan has also proved to be useful during diffraction screening of crystallization trays, where refraction effects from both the crystallization well and the surface of the drop can mean that crystals appear to be tens of micrometres away from their true locations.

Work is currently ongoing to combine the results of grid scans at multiple φ angles for automated alignment and tracking of multiple crystals held in a loop and also in the development of grid scanning utilizing the readout of the fluorescence detector, as this allows lower dose grid scans for crystals containing an anomalous scatterer. Future work will allow users to select areas of arbitrary shape, thus minimizing data collection over empty regions of space or sample-mount areas that are not of interest.

The slow scan is in routine use on all other MX beamlines at Diamond Light Source and automated X-ray crystal centring using this method is currently being implemented. The precision requirement of sample positioning on MX beamlines is typically dictated by the beam size and the crystal size used. The requirements for grid scanning in general are similar to those for standard crystallographic data collection and are therefore related to the beam size being used. This makes the implementation of grid scanning appropriate for many MX beamlines, not only microfocus beamlines, and it should not require special instrumentation.

In all cases a grid scan can provide an undistorted view that is not possible with visible light and presents an opportunity for both rapid and fully automated screening of crystallization trays and diffraction-based alignment of microcrystals.

## References

[bb1] Beteva, A. *et al.* (2006). *Acta Cryst.* D**62**, 1162–1169. 10.1107/S0907444906032859 17001093

[bb2] Caffrey, M. (2000). *Curr. Opin. Struct. Biol.* **10**, 486–497. 10.1016/s0959-440x(00)00119-6 10981640

[bb3] Cherezov, V., Hanson, M. A., Griffith, M. T., Hilgart, M. C., Sanishvili, R., Nagarajan, V., Stepanov, S., Fischetti, R. F., Kuhn, P. & Stevens, R. C. (2009). *J. R. Soc. Interface*, **6**, S587–S597. 10.1098/rsif.2009.0142.focus PMC2843980 19535414

[bb4] Evans, G., Alianelli, L., Burt, M., Wagner, A. & Sawhney, K. (2006). *AIP Conf. Proc.* **879**, 836–839.

[bb5] Hülsen, G., Broennimann, C., Eikenberry, E. F. & Wagner, A. (2006). *J. Appl. Cryst.* **39**, 550–557.

[bb6] Ji, X., Sutton, G., Evans, G., Axford, D., Owen, R. L. & Stuart, D. (2010). *EMBO J.* **29**, 505–514. 10.1038/emboj.2009.352 PMC2824454 19959989

[bb7] Riekel, C., Burghammer, M. & Schertler, G. (2005). *Curr. Opin. Struct. Biol.* **15**, 556–562. 10.1016/j.sbi.2005.08.013 16168633

[bb8] Song, J., Mathew, D., Jacob, S. A., Corbett, L., Moorhead, P. & Soltis, S. M. (2007). *J. Synchrotron Rad.* **14**, 191–195. 10.1107/S0909049507004803 17317920

[bb9] Zhang, Z., Sauter, N. K., van den Bedem, H., Snell, G. & Deacon, A. M. (2006). *J. Appl. Cryst.* **39**, 112–119.

